# Lumbar Spinal Candida Glabrata Treated Without Surgical Intervention: A Case Report

**DOI:** 10.7759/cureus.1371

**Published:** 2017-06-20

**Authors:** Ryan M Schiedo, William Lavelle, Mike H Sun

**Affiliations:** 1 Medical Student, Suny Upstate Medical University, Syracuse, NY; 2 Department of Orthopedic Surgery, Suny Upstate Medical University, Syracuse, NY

**Keywords:** candida glabrata, vertebral osteomyelitis, low back pain, micafungin, osteoarticular pain, infection, non-surgical treatment

## Abstract

Candida glabrata is a low virulent commensal fungal organism that, rarely, can cause osteomyelitis. Diagnosis of such an infection is often difficult as the case typically presents with an insidious onset of back pain and minimally elevated biomarkers of inflammation. Furthermore, it is difficult to eradicate and often resistant to common antifungals.

A 61-year-old man presented with an eight-month history of persistent low back pain which had unsuccessfully been managed by his primary care physician. He had a past surgical history of gastric by-pass complicated by adhesions, ulceration, and perforation with an infection of Candida glabrata that had been treated with intravenous micafungin. Radiological examination showed degenerative changes with suspicion of osteomyelitis and a computerized tomography (CT)-guided biopsy provided tissue samples with subsequent positive cultures for Candida glabrata. The patient was admitted for fungal osteomyelitis with Candida glabrata, treated with intravenous micafungin, and his infection was resolved after six months. At two-year follow-up his back pain has been resolved and no infection was present. In a patient with osteoarticular pain and a previous history of candidal infection with possible candidemia, one should maintain suspicion for fungal osteomyelitis.

## Introduction

Candida glabrata is a low virulent commensal fungal organism present on human mucosal tissues [1]. Candida glabrata is a rare opportunistic pathogen known to cause osteomyelitis. Diagnosing Candida glabrata osteomyelitis is challenging, as patients with lumbar osteomyelitis typically present with an insidious onset of back pain and minimally elevated biomarkers of inflammation [2].  It is paramount to accurately identify Candida glabrata from other candida species, as it has an increased natural resistance to antifungal agents, making successful treatment difficult [3].

## Case presentation

Informed consent was obtained from the patient. We saw a 61-year-old man with an eight-month history of persistent low back pain that woke him from sleep. He denied numbness, tingling, and bowel and bladder incontinence. He also denied any history of fever, chills, cough, vomiting, diarrhea, or night sweats. On a visual analog scale (VAS), he ranked his pain 6/10. His primary care physician managed his chronic pain unsuccessfully with hydrocodone-acetaminophen, a cane, and physical therapy. 

His past medical history comprised depression, hypertension, anxiety, and paranoid schizophrenia, but was negative for HIV and immunodeficiency. His surgical history included a Roux-en-Y gastric by-pass performed 10 months ago, complicated by adhesions, ulceration, and perforation. An exploratory laparotomy was performed at that time and an entero-cutaneous fistula was discovered. It was infected with Candida glabrata which was later found to be partially resistant to fluconazole. The fistula was repaired and he was treated with intravenous micafungin over the course of several weeks.

On physical examination, his lumbar range of motion with twisting and bending was painful at end range. Palpation of the spinal processes and paraspinal region elicited tenderness. His neurological examination was unremarkable. Lab values: white blood cell count (WBC), 8,700 cells/mm^3^ with 68% neutrophils, 20% lymphocytes, 8% monocytes, 3% eosinophils, and 1% basophils; hemoglobin (Hb), 12.8 g/dL; hematocrit, 37.8%; platelets 212,000/mm^3^; erythrocyte sedimentation rate (ESR), 31 mm/hr; C-reactive protein (CRP), 13.2 mg/L; and liver function tests were within normal limits.

**Figure 1:**
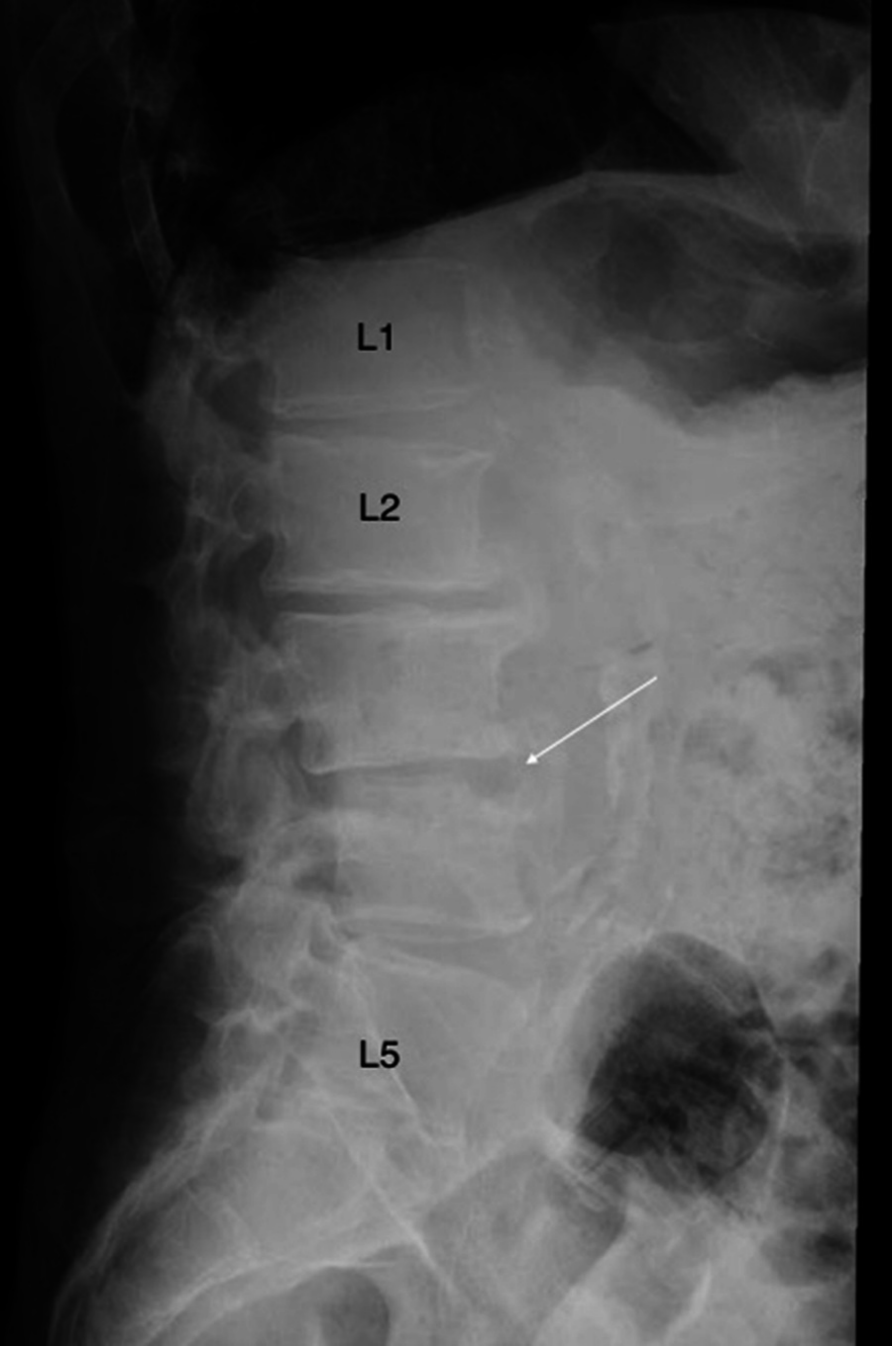
Lateral X-ray of the lumbar spine. There is evidence of erosive change on the anterior and superior plate of L4 that was not previously present. There is loss of disc height at L3-L4 and grade 1 anterolisthesis of L4-L3, findings suggestive of osteomyelitis and discitis.

**Figure 2:**
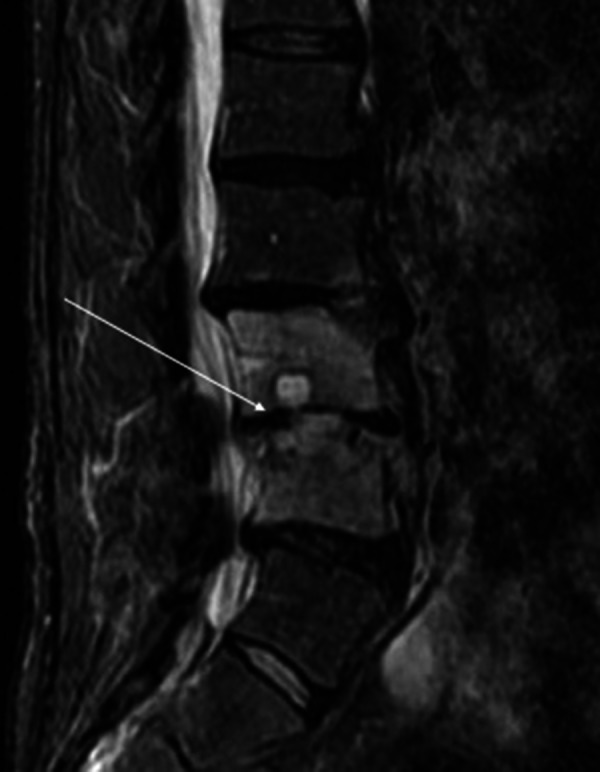
Non-contrast T2 MRI of the spine. There is evidence of osteomyelitis at the L2-L3 and L3-L4 intervertebral levels with abscess in the disc space at the L3-L4 and anterior aspect of the disc at L2-L3.

Plain radiographs revealed degenerative changes without evidence of fracture (Figure 1). Magnetic resonance imaging (MRI) raised the suspicion of osteomyelitis involving L2-3 and L3-4 with a possible abscess in the L3-4 disk space (Figure 2). A computerized tomography (CT)-guided biopsy provided tissue which registered positive cultures for Candida glabrata resistant to fluconazole, itraconazole, and posaconazole, but sensitive to micafungin and amphotericin B. No anaerobes, acid-fast bacilli, or aerobic organisms were present in the bioptic material.

**Figure 3:**
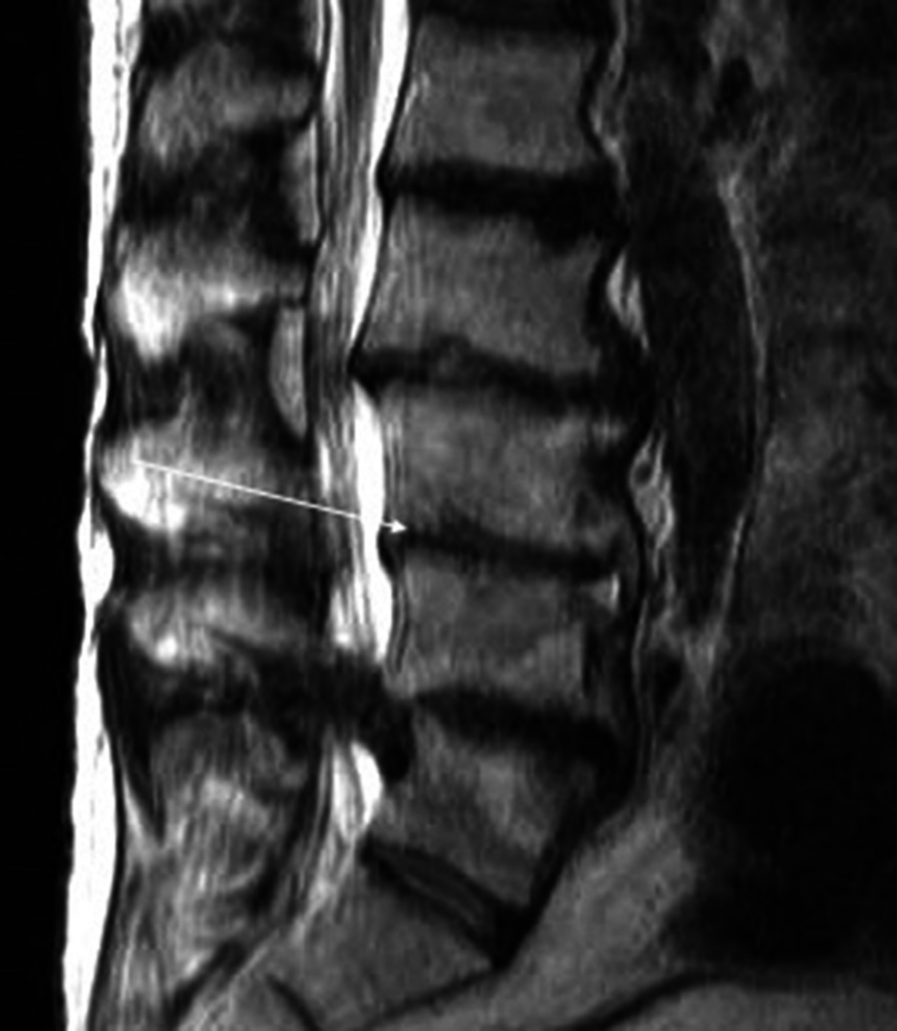
MRI without contrast. This image was taken nearly a year after he was discharged from the hospital. The anterior edema in the endplate is no longer present on the T2 MRI scan. The patient is asymptomatic with no signs of infection.

The patient was admitted for fungal osteomyelitis with Candida glabrata and treatment with intravenous micafungin was begun. After six months of treatment, his infection resolved and he was discharged. At two-year follow-up, his back pain has fully resolved (VAS Analog Scale 1/10) and an MRI revealed resolution of the infection (Figure 3).

## Discussion

Osteomyelitis with Candida glabrata is a rare infection that is difficult to treat. The most commonly identified portal of entry for Candida glabrata fungemia is through the abdomen [4], a finding consistent with our case.  His initial abdominal infection grew Candida glabrata partially resistant to fluconazole. Ten months later, a biopsy of his lumbar spine showed that the organism was fully resistant to fluconazole, itraconazole and posaconazole. It is likely that the vertebrae were seeded at the time of the initial Candida glabrata infection.

Identification of fungal osteomyelitis can be difficult because sensitive and specific biomarkers are lacking for this infection. An elevated ESR is the most common laboratory abnormality reported in patients with candidal vertebral osteomyelitis. In a study of 207 patients with fungal osteomyelitis, 87% of patients had elevated ESR [5]. Our patient had a moderately elevated ESR and CRP, but lacked an elevated WBC or fever. The lack of sepsis warranted a search for a less common pathogen. In our case, the first biopsy grew Candida glabrata; however, after reviewing the literature it was found  this is not always the case. Miller and Mejicano suggested that if the first biopsy is negative, but there is a high suspicion for osteomyelitis, then a second biopsy should be performed; and if that is also negative, surgical exploration should be undertaken [5]. Identifying the organism is paramount, as therapy for mycobacterial and fungal osteomyelitis are vastly different.

The primary pathophysiological mechanism of vertebral osteomyelitis in adults is hematogenous dissemination [6].  When infection is spread hematogenously, the most frequent site of bony involvement is the lumbar spine [7].  Patients typically have an effusion and tenderness with joint range of motion [8]. All of these findings were consistent with our case.

## Conclusions

Diagnosing candida osteomyelitis is difficult as it is an uncommon pathological organism. When a patient has osteoarticular pain and a previous history of candidemia, one should maintain suspicion for a subsequent osteomyelitis. This holds true even if the initial infection was months or years ago. Culture and antibiogram sensitivities should be performed to ensure proper treatment of the infection.
